# Using dapagliflozin to reduce symptoms in transthyretin cardiac amyloidosis: An n-of-1 trial series pilot study

**DOI:** 10.1371/journal.pone.0346065

**Published:** 2026-10-07

**Authors:** Erik Håkansson, Intissar Anan, Mattias Brunström, Karin Hellström-Ängerud, Per Lindqvist, Björn Pilebro, Jonas Wixner, Krister Lindmark

**Affiliations:** 1 Department of Public Health and Clinical Medicine, Umeå University, Umeå, Sweden; 2 Department of Nursing, Umeå University, Umeå, Sweden; 3 Department of Diagnostics and Intervention, Umeå, Sweden; 4 Karolinska Institute, Department of clinical sciences, Stockholm, Sweden; All India Institute of Medical Sciences - Jodhpur, INDIA

## Abstract

**Background:**

The role of guideline-directed medical therapy for heart failure (HF) is unclear in transthyretin amyloid cardiomyopathy (ATTR-CM). Dapagliflozin has demonstrated beneficial effects across HF phenotypes, and retrospective studies suggest potential benefit in ATTR-CM; however, prospective data are lacking. This pilot study evaluated the effect and tolerability of dapagliflozin in patients with ATTR-CM to inform the design of a larger randomized trial.

**Methods:**

Participants were followed for 30 weeks over three study periods: 8 weeks baseline without study medication, 12 weeks intervention with dapagliflozin 10 mg once daily, and 10 weeks withdrawal without study medication. The study was open-label and nonrandomized. Vital signs and NT-proBNP were assessed every second week, while Kansas City Cardiomyopathy Questionnaire total symptom score (KCCQ-TSS) and 6-minute walking test (6MWT) were assessed every 4 weeks. Results were analyzed using linear mixed-effects models.

**Results:**

Seven of ten enrolled patients completed the trial. One participant discontinued because of genitourinary adverse effects, and two withdrew for reasons unrelated to the intervention. The estimated intervention-versus-baseline difference was −142 pg/mL (95% CI −324–41) for NT-proBNP, 24.3 m (95% CI −2.8 to 51.5) for 6MWT distance, and 0.4 points (95% CI −4.0 to 4.8) for KCCQ-TSS. Sitting SBP was 8.8 mmHg lower during intervention than during baseline (95% CI −13.2 to −4.4). No increase in hypotensive symptoms or cardiovascular adverse events was detected.

**Conclusions:**

Dapagliflozin was generally well tolerated in this pilot study of patients with ATTR-CM. The estimated differences in NT-proBNP, 6MWT distance, and KCCQ-TSS were imprecise and did not provide conclusive evidence of treatment effects. SBP was lower during treatment, without a detected increase in hypotensive symptoms. These exploratory findings support further evaluation in an adequately powered randomized trial.

**Trial registration:**

This study was registered with clinical trial number EudraCT no 2021-003674-32

## Introduction

Transthyretin amyloid cardiomyopathy (ATTR-CM) is a deadly and debilitating disease caused by an accumulation of misfolded transthyretin (TTR) in the myocardium [1]. The amyloid infiltration causes an expansion of extracellular volume, myocyte necrosis and increased tissue stiffness, which results in a hypertrophic and restrictive cardiomyopathy with heart failure (HF) and a high occurrence of cardiac arrhythmias such as atrial fibrillation and atrioventricular block [1,2]. Prognosis is poor after diagnosis, with a median survival of only 2.5–3.5 years.

Transthyretin amyloidosis (ATTR) can occur both due to variants in the *TTR* gene (ATTRv) and with normal, wild-type TTR (ATTRwt) [3]. ATTR-CM has historically been considered a rare disease, but advancements in non-invasive diagnostic techniques and the introduction of disease modifying treatment have led to higher case-finding rates [4].

Conventional HF drugs offer uncertain benefit and may be associated with harm in these patients [5,6]. Patients usually receive diuretic therapy with loop-diuretics or mineralocorticoid receptor antagonists (MRA) to control volume overload [7]. Currently, the TTR-stabilizers tafamidis and acoramidis are approved for treatment of ATTR-CM [8,9]. TTR-stabilizers improve survival and slow decline in functional capacity, but the benefit is uncertain in advanced disease and it is not universally subsidized or reimbursed due to high cost [10]. Additionally, TTR-stabilizers do not improve already existing HF symptoms, so for symptomatic ATTR-CM patients and especially those with advanced disease not eligible for TTR-stabilizers there is an unmet need of proven pharmacotherapies.

Sodium-glucose cotransporter 2 inhibitors (SGLT2i) have proven effective in improving clinical outcomes and alleviating HF symptoms, irrespective of left ventricular ejection fraction (LVEF) [11–14], and have quickly been established as a cornerstone treatment for most patients with HF [15], including those with preserved ejection fraction [16]. While amyloid cardiomyopathy can present with reduced LVEF, most patients present as HF with preserved EF (HFpEF), and a significant proportion of HFpEF could be secondary to amyloid cardiomyopathy [4]. SGLT2 inhibitors may alleviate symptoms in amyloid cardiomyopathy through osmotic diuresis, reduced plasma volume, and lower cardiac filling pressures, potentially unloading the restrictive, amyloid-infiltrated ventricle. Additional proposed mechanisms include improved vascular compliance, erythropoiesis, iron metabolism, and myocardial energy utilization.

Patients with known ATTR-CM were excluded from all major SGLT2 inhibitor trials. The mechanisms through which SGLT2 inhibitors improve cardiac function and clinical outcomes remain under investigation. Several retrospective studies indicate that SGLT2 inhibitors are generally tolerated and may be beneficial in ATTR-CM [17–21]. To our knowledge, no prospective studies have investigated this question. Evidence of benefit or harm would be clinically relevant in a population with few established symptomatic treatment options.

This pilot study aims to prospectively investigate the tolerability of the SGLT2i dapagliflozin treatment in patients with ATTR-CM and its effect on N-terminal pro-brain natriuretic peptide (NT-proBNP), HF symptoms, walking distance on a 6-minute walking test (6MWT) and estimates of filling pressures from transthoracic echocardiograms (TTE), to inform the feasibility and design of a possible larger trial in the future.

## Methods

### Trial design

This study was conducted as a series of open-label, single-crossover, n-of-1 trials with three phases: an 8-week baseline period, a 12-week intervention period and a 10-week withdrawal period. This design was chosen to maximise the information yield from a small number of participants in a time- and cost-efficient manner.

A single-crossover design was chosen to balance the risk of confounding from the progressive nature of the disease being studied, patient exertion, and cost. No washout period was included between baseline and intervention. The 10 week withdrawal phase after was considered sufficient for elimination of dapagliflozin, which has a reported elimination half-life of approximately 12.9 hours after oral administration of 10 mg. The main pharmacodynamic effect, urinary glucose excretion, is generally expected to persist for approximately 3 days after discontinuation, although longer persistence of glucosuria and/or ketoacidosis has been reported in some post-marketing cases. Thus, the 10-week withdrawal period was considered adequate for resolution of direct drug-mediated SGLT2 inhibition. However, the duration of potential downstream physiological effects is uncertain.

### Patient selection

The primary inclusion criteria required patients to have HF with New York Heart Association (NYHA) Class II-IV due to ATTR-CM, diagnosed with either biopsy or Perugini grade II or III uptake on a ^99m^Tc-DPD (Technetium-99 difosfonopropandicarboxylic acid) scintigraphy with lack of signs of amyloid light chain amyloidosis [22], and optimal medical therapy for at least 4 weeks according to treating physician. Exclusion criteria included current treatment with SGLT2i or a history of intolerance to SGLT2i, insulin-treated diabetes mellitus, enrolment in another clinical trial, or inability or unwillingness to comply with study procedures. For a full list of inclusion and exclusion criteria, see trial protocol in Supplement 1.

Patients were opportunistically pre-screened at the outpatient clinic at a single centre, Umeå University Hospital. Patients who met eligibility criteria were informed of the trial and asked about their interest in participating. Those who expressed interest were provided with a patient information letter and their interest was followed up via a phone call. If patients were still interested after reading the information letter, they were booked for a screening visit, where the final eligibility assessment was made, and informed consent was collected. The first study visit was scheduled to occur within 4 weeks of the screening visit. The study protocol was designed to minimize risks for patients with frequent monitoring and insulin treated diabetes was an exclusion criterion to reduce the risk for euglycemic ketoacidosis. See study protocol for details.

Our goal was to enrol 10 patients in the trial.

### Ethics

This study was registered with clinical trial number EudraCT no 2021-003674-32. This study was approved by the Swedish Ethical Review Authority (file number 2022-05768-01) and the Swedish Medical Products Agency (file number 5.1–202288111). The trial is compliant with the Declaration of Helsinki, and all subjects were given oral and written information of the study and gave oral and written permission to participate in the study. First patient in was included 17 April 2023 and the end of the trial was 23 May 2025

### Intervention

Dapagliflozin 10 mg once daily in the morning. Dapagliflozin was initiated the day after the last baseline period visit and then stopped at the last intervention period visit.

### Outcomes

At every visit, blood samples were collected, vital parameters were recorded, and weight was measured. Analyses on blood samples included levels of n-terminal natriuretic peptide type B (NT-proBNP), blood count, and creatinine. Vital parameters included sitting blood pressure after at least five minutes of rest, as well as measurements after three minutes of standing. Every second visit, patients performed a 6-minute walking test (6MWT) and filled in a Kansas City Cardiomyopathy Questionnaire (KCCQ). At the last visit of every phase, a transthoracic echocardiogram (TTE) was performed. The exploratory efficacy outcomes were chosen as clinically relevant surrogate markers which may be influenced by other factors. All efficacy endpoints are to be considered exploratory and surrogates for clinical outcomes.

The predefined primary outcome was the difference in mean NT-proBNP level or slope between study periods. Secondary outcomes were differences in mean KCCQ total symptom score (TSS) and 6MWT distance, or their slopes, between periods. Analyses compared periods within participants. Key safety endpoints were sitting and standing blood pressure, diuretic use, haemoglobin concentration, creatinine, and body weight. The protocol did not specify numerical safety thresholds for terminating the intervention; this decision was left to the investigator. Preplanned exploratory endpoints were differences in echocardiographic estimates of filling pressure [23].

### Outcome assessment tools

NT-proBNP is a peptide produced in the heart in response to increased wall tension. It is widely used in HF diagnosis and follow-up, and higher levels are associated with more severe symptoms, worse prognosis and higher filling pressures [24]. It is used in the UK National Amyloidosis Centre scale for staging of ATTR-CM [25]. NT-proBNP levels are influenced by factors such as renal function, volume status, and biological variability. Samples were analyzed with a Cobas 8000 machine and proBNP II STAT reagents (Roche Scandinavia).

KCCQ is a 23-item questionnaire that measures health-related quality of life, and is well validated in patients with HF. It has been translated into many languages, including Swedish which was the version used in this study. Answers are transformed into several sub scores, including total symptom score (TSS), overall summary score (OSS) and clinical summary score (CSS), each ranging from 0–100 points with a higher score meaning less symptoms [8,26,27].

6MWT is a test to evaluate a patient’s physical exercise capability by measuring how far they can walk in 6 minutes by walking back and forth over a 30-meter distance. It is correlated with aerobic exercise capacity and has prognostic implications, but is susceptible to interference from non-cardiopulmonary walking limitations, e.g., neuropathy and also to severe pulmonary disease. It is also susceptible to performance bias in the open-label design [28,29].

### Statistical methods

The results were analyzed using a mixed effects linear model. The model included study period as a fixed effect, and study participant as a random effect, expressed 𝑦_𝑖𝑗_ = 𝛽_0_ + 𝛽_x𝑖𝑗_ + 𝑢_𝑖_ + *ε*_𝑖𝑗_ where *y* denotes the continuous outcome variable (i.e., NT-proBNP); *x* is the period (baseline, intervention, withdrawal); *i* is the individual and *j* is the longitudinal visits. 𝛽_0_ is the estimated outcome level in baseline period; 𝛽_x_ the estimated difference in outcome levels between different periods and the baseline; 𝑢 is the normally distributed random intercept for each individual; *ε* is the random error. A first order autoregressive covariance structure was assumed. Results are reported as the difference in estimated marginal means for each period compared to the baseline period with 95% confidence intervals (CI). The mixed-effects models were fitted using maximum likelihood estimation and included all available observations without imputation. This approach assumes that missing data are missing at random, conditional on the observed data included in the model. The extent and reasons for missing outcome data were assessed descriptively.

The statistical analysis plan (Supplement 2) prespecified a piecewise linear mixed-effects model to estimate differences in slopes between study periods. After model fitting, the number of parameters was judged excessive relative to the sample size, resulting in unstable estimates and very wide confidence intervals. We therefore used estimated mean differences between periods for the main descriptive analysis. This was a post hoc, justified deviation from the prespecified statistical analysis plan. Given the small number of participants, the random intercept was included primarily to account for within-participant correlation rather than to provide a precise estimate of between-participant variability.

Results from the prespecified piecewise slope model are reported in Supplementary Table 1 for transparency and are interpreted descriptively.

All analyses were performed in IBM SPSS Statistics version 29.

Participants who concluded at least 2 study periods (baseline + intervention) were included in the analysis.

Since this was a pilot study, no power- or sample size calculations were performed, and results of statistical analysis are shown for descriptive purposes without p-values. All outcomes should be considered exploratory.

## Results

### Study population

A total of 54 patients were pre-screened, of which 14 met eligibility criteria. Reasons for ineligibility were current participation in other trials that did not allow concurrent enrolment (n = 9), other cause for heart failure (n = 8), already on SGLT2i treatment (n = 6), NYHA-class 1 (n = 6), inability to visit study site due to residing too far away from study site (n = 5), insulin treated diabetes mellitus (n = 2), Karnofsky performance status <60 (n = 1), eGFR < 25 ml/1.73m[2]/min (n = 1), expected survival < 9 months (n = 1), and declined further information (n = 1).

Of the 14 patients who received the information letter, 10 consented to screening and were enrolled. Three participants did not complete the trial, corresponding to 30% attrition. Two withdrew during baseline before receiving dapagliflozin: one because the travel required for study visits was considered too burdensome and one because of non-adherence to study visits and concomitant medication. One participant discontinued at the start of intervention because of worsening lower urinary tract symptoms (Fig 1).

**Fig 1:**
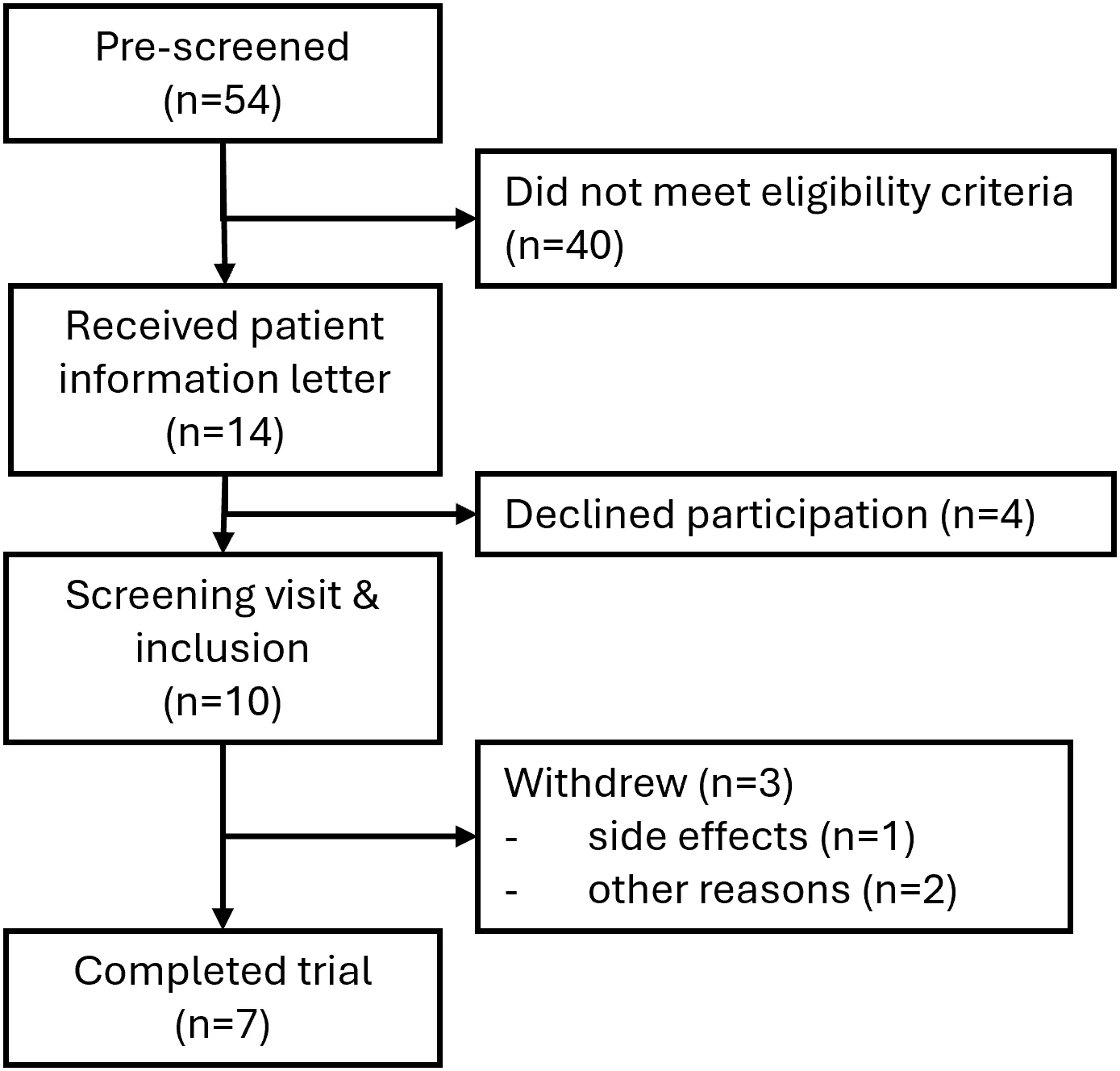
Patient inclusion flowchart.

Baseline characteristics of the seven participants who completed the trial and the three who did not complete the trial are shown in Table 1. Among completers, mean age was 78 years, two participants were female, five were in NYHA class II, and two were in NYHA class III. Four had wild-type ATTR-CM and three had variant ATTR-CM with the Val30Met genotype.

**Table 1:** Baseline characteristics of study completers and non-completers.

Characteristic	Completed study (n = 7)	Did not complete study (n = 3)
Sex: male/female	5/2	2/1
Age, years, mean (SD)	78 (6.6)	78.7 (3.8)
BMI, kg/m², mean (SD)	27.4 (3.3)	25.6 (2.6), n = 2
NYHA class II/III	5/2	2/1
eGFR, mL/min/1.73 m², mean (SD)	58 (7.1)	53.0 (18.2)
NT-proBNP, pg/mL, median (range)	1261 (931–2320)	2034 (1182–5052)
Haemoglobin, g/L, mean (SD)	137 (20)	113.3 (16.2)
Heart rate, bpm, mean (SD)	73 (10)	68.7 (9.0)
Sitting SBP, mmHg, mean (SD)	130.7 (8.7)	136.0 (23.1)
Sitting DBP, mmHg, mean (SD)	78.4 (6.9)	80.3 (9.5)
Standing SBP, mmHg, mean (SD)	145.4 (9.3)	148.7 (16.5)
Standing DBP, mmHg, mean (SD)	83.8 (7.9)	81.7 (7.2)
Troponin T, ng/L, mean (SD)	44 (16.9)	53.3 (8.4)
Atrial fibrillation: paroxysmal/permanent	2/3	0/2
Pacemaker, n	3	1
Diabetes mellitus, n	0	0
Hypertension, n	3	2
Polyneuropathy, n	2	Not recorded
Genotype: wild-type/Val30Met	4/3	3/0
IVSD, mm, mean (SD)	17.9 (1.2)	17.0 (2.6)
Perugini grade II/III	2/5	3/0
Years since diagnosis, mean (SD)	1.9 (1.1)	3.3 (2.5)
Tafamidis, n	3	2
NOAC, n	5	2
MRA, n	7	2
ARB or ACEi, n	2	0
Beta-blocker, n	2	1
Loop diuretic, n	6	2

Data are n, mean (SD), or median (range), as indicated. No formal comparisons were performed. BMI was missing for one non-completer. Polyneuropathy status was not recorded for non-completers. SBP, systolic blood pressure; DBP, diastolic blood pressure; eGFR, estimated glomerular filtration rate; IVSD, interventricular septal thickness in diastole; NOAC, non-vitamin K oral anticoagulant; MRA, mineralocorticoid receptor antagonist; ARB, angiotensin receptor blocker; ACEi, angiotensin-converting enzyme inhibitor.

The observed baseline characteristics of non-completers are reported alongside those of completers in Table 1; individual data for non-completers are provided in Supplementary Table 2. No formal between-group comparisons were performed. Of the four patients who received the invitation letter but declined further participation, three were in NYHA class III.

### Outcomes

Individual trends for NT-proBNP, KCCQ-TSS, 6MWT, and sitting systolic blood pressure are shown in Fig 2.

**Fig 2:**
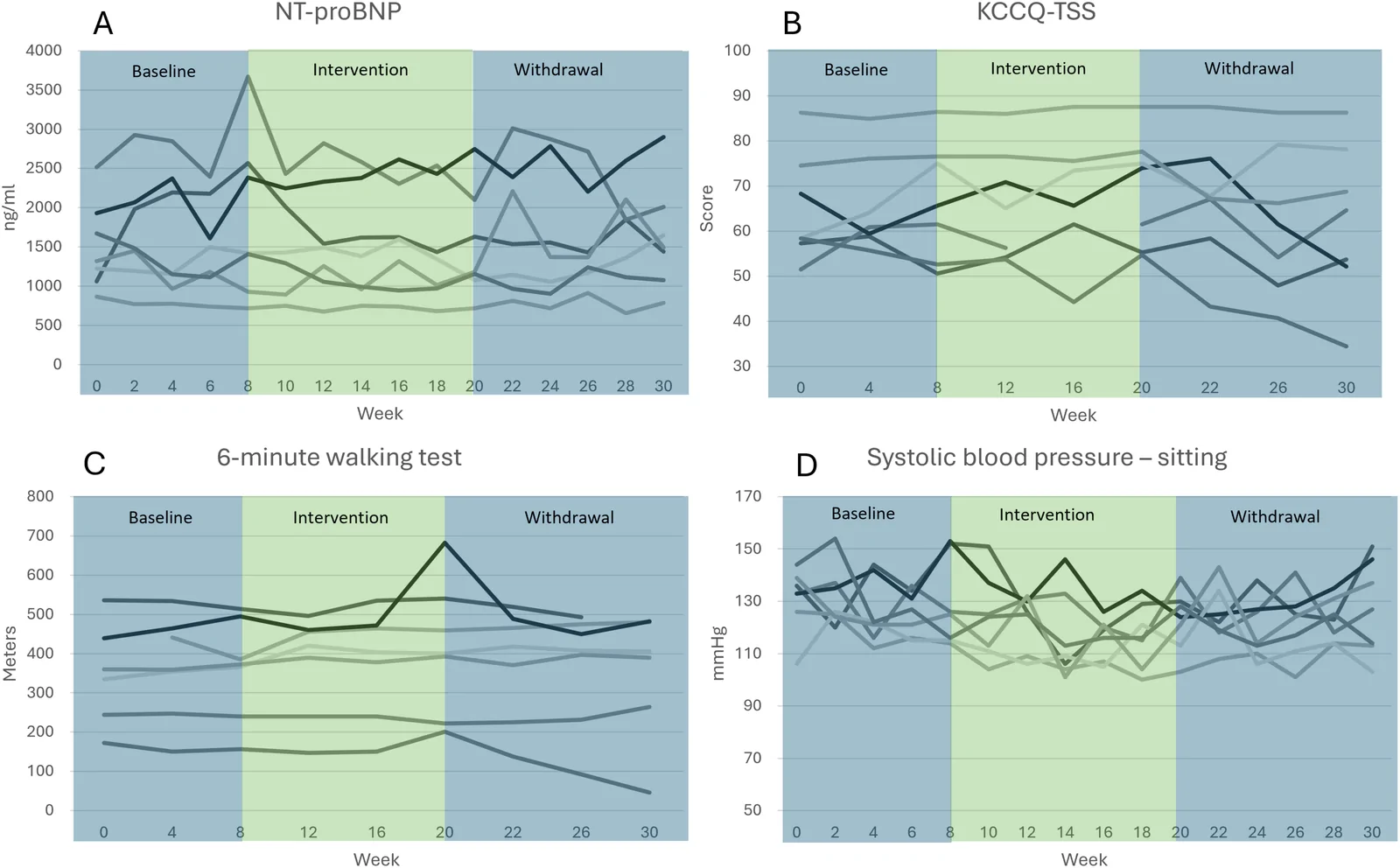
NT-proBNP, N-terminal pro-brain natriuretic peptide; KCCQ-TSS, Kansas City Cardiomyopathy Questionnaire- Total Symptom Score. A: Individual trends for NT-proBNP per week. B: Individual trends for KCCQ-TSS. There is one missing value at week 16. C: Individual trends for 6MWT. There are 3 missing values: one at week 0, one at week 30 and one at week 26. The missing value at week 26 is interpolated for visualization purposes. D: Individual trends for systolic blood pressure measured in the sitting position.

Among the seven participants included in the analysis, NT-proBNP, blood pressure, body weight, haemoglobin, and creatinine measurements were complete across study visits. Missing data occurred only for KCCQ-TSS and 6MWT. For KCCQ-TSS, one observation was missing at week 16 because the questionnaire was incompletely completed. For 6MWT, three observations were missing: one at week 0 because of incorrectly entered data, one at week 26 because the participant was unable to perform the test due to general fatigue, and one at week 30 because of a temporary non-cardiac impediment. Thus, missingness was limited in extent and appeared to be related to procedural issues or temporary clinical circumstances rather than to treatment response or the observed outcome values.

The estimated differences between study periods are shown in Table 2. For NT-proBNP, KCCQ-TSS, and 6MWT distance, the 95% confidence intervals included zero and did not provide statistically conclusive evidence of differences between intervention and baseline. Sitting and standing SBP and DBP were lower during intervention than during baseline, with confidence intervals that excluded zero.

**Table 2:** Mean change between study periods.

	Intervention vs baseline	Withdrawal vs intervention
Primary and secondary outcomes		
NT-proBNP – pg/mL (95% CI)	−142 (−324 – + 41)	+129 (−53 – + 312)
KCCQ-TSS – points (95% CI)	+0.4 (−4.0 – + 4.8)	−2.9 (−7.3 – + 1.5)
6MWT – meters (95% CI)	+24.3 (−2.8 – + 51.5)	−22.1 (−49.3 – + 5.1)
Safety outcomes		
SBP, sitting – mmHg (95% CI)	−8.8 (−13.2 – −4.4)	+3.2 (−1.3 – + 7.6)
DBP, sitting – mmHg (95% CI)	−4.1 (−6.7 – −1.5)	−0.7 (−3.3 – + 1.9)
SBP, standing – mmHg (95% CI)	−6.5 (−10.8 – −2.2)	+2.0 (−2.3 – + 6.3)
DBP, standing – mmHg (95% CI)	−3.9 (−6.3 – −1.5)	−1.1 (−3.5 – + 1.3)
Weight – kg (95% CI)	−1.1 (−1.7 – −0.5)	+0.8 (0.2–1.4)
Haemoglobi*n – g/l* (95% CI)	+4.6 (+1.2 – + 8.6)	−5.2 (−8.6 – −1.7)
Creatinine – µmol/l (95% CI)	+1.4 (−1.5 – + 4.3)	−1.3 (−4.2 – + 1.6)
Exploratory outcomes		
LV Strain - % (95% CI)	−0.2 (−1.4 – + 0.9)	+0.2 (−0.9 – + 1.4)
E/e’ (95% CI)	+0.1 (−3.2 – + 3.4)	+0.6 (−2.7 – + 4.0)
TR Vmax – m/s (95% CI)	+0.02 (−0.2 – + 0.2)	+0.1 (−0.1 – + 0.3)

NT-proBNP, N-terminal pro-brain natriuretic peptide; KCCQ-TSS, Kansas City Cardiomyopathy Questionnaire Total Symptom Score; 6MWT, 6-minute walking test; LV, Left Ventricle; TR Vmax, Tricuspid regurgitation maximal velocity; SBP, Systolic Blood Pressure; DBP, Diastolic blood pressure; CI, Confidence interval.

There were minor differences in TTE-derived parameters without a coherent pattern.

In a post hoc sensitivity analysis excluding the influential but verified week-20 6MWT observation, the estimated intervention-versus-baseline difference was 12.5 m (95% CI −6.7 to 31.8), compared with 24.3 m (95% CI −2.8 to 51.5) in the primary analysis. Results from the exploratory model including period-specific slopes are presented in Supplementary Table 1. Estimates from this model were imprecise and should be interpreted cautiously.

### Safety

There were no serious adverse events during the trial. Two patients reported increased urinary volumes during the intervention period, and one patient discontinued dapagliflozin and withdrew from the study because of worsening lower urinary tract symptoms.

There was a reduction in blood pressure, both sitting and standing, during the intervention period which partially recovered during the withdrawal period (Fig 2 and Table 2). This was not associated with an increase in orthostatic symptoms.

There was a reduction in body weight during the intervention period which recovered during the withdrawal period. Haemoglobin concentration increased during the intervention period and decreased during the withdrawal period.

During the intervention phase, 2 patients stopped taking oral furosemide; they later restarted furosemide in the withdrawal phase due to ankle oedema. One patient temporarily increased dose of furosemide during 4 weeks in the intervention phase and later increased the dose again in the beginning of the withdrawal phase until the end of the trial due to worsening ankle oedema. No other treatment changes were recorded during the trial period.

## Discussion

In this small exploratory study, the point estimates for NT-proBNP and 6MWT favored the intervention period, but the confidence intervals included zero and the findings were not statistically conclusive. KCCQ-TSS did not show a clearly detectable difference between baseline and intervention. In a recent meta-analysis of non-ATTR-CM heart failure, SGLT2 inhibitors were associated with an NT-proBNP difference of −136 pg/mL, a KCCQ-TSS difference of 2.88 points, and a 6MWT difference of 23.98 m [30]. These estimates are close to the point estimates in the present study, but the small sample prevents reliable comparison.

There was a decrease in blood pressure during the intervention period that was quite a bit larger than expected. A meta-analysis of 3 RCTs in patients with HF comparing dapagliflozin to placebo found a mean difference of only 1 mmHg [31]. The reduction in blood pressure appears to be larger in patients with type 2 diabetes mellitus and hypertension as described in a meta-analysis by Zhang et al [32], where across 8 RCTs the mean reduction was 4.5 mmHg. However, the patients in this trial were non-diabetic and normotensive, so this may point to an interaction with the underlying pathology of ATTR-CM. A common issue for patients with ATTR-CM is intolerance to blood pressure lowering and orthostatic blood pressure falls. Although there was no increase in hypotensive symptoms in this trial, the comparatively large decrease in blood pressure might warrant caution with SGLT2i treatment in patients with ATTR-CM, especially the ones with pre-existing hypotensive symptoms.

There are several observational and retrospective studies assessing SGLT2i in ATTR-CM. The largest so far (Porcari et al 2024) compares 220 patients with ATTR-CM to 220 propensity score matched controls with ATTR-CM, selected from a longitudinal observational study cohort [19]. They report a substantially lower risk of cardiovascular mortality for SGLT2i-treated patients with a hazard ratio of 0.41 (95% CI 0.24–0.71), lower rates of initiation of loop diuretics, slower rise in NT-proBNP over time, and no significant difference in blood pressure after 12 months compared to baseline. The mortality reduction is larger than what is reported in RCTs with SGLT2i and HF, where the HR has repeatedly been found to be between 0.82 and 0.92 [11–14], which may indicate the effect is exaggerated in the observational study. Their observations on NT-proBNP and diuretic use is compatible with our findings, while their observations on blood pressure is not.

ATTR-CM is ultimately a disease driven by accumulation of amyloid deposits and the only interventions that have improved outcomes in RCTs are targeted at the amyloid deposition process [8,9]. Dapagliflozin has no known interaction with TTR or amyloidogenesis, so any treatment effect is unlikely to be due to disease modification. This should theoretically make it unlikely to find a large mortality reduction with dapagliflozin-treatment, while an effect on symptoms, functioning capacity and decompensation risk is more likely. Current theories of the beneficial effects of SGLT2i in HF include decongestion due to reduced plasma volume [33], improved vascular compliance [34], increased erythropoiesis [35], improved cardiac energy utilization [36], and improved iron metabolism [37], all of which could be potentially beneficial to the amyloid-afflicted heart. The choice of dapagliflozin over empagliflozin was simply due to dapagliflozin being more commonly used at our clinic.

Based on retrospective and observational data [17–20], SGLT2i are already recommended in the treatment of ATTR-CM by some [38]. Our findings do not discourage that, but clinical benefit should be demonstrated in an adequately powered RCT.

### Limitations

This pilot study was designed for 10 participants without a formal power or sample-size calculation, and only seven completed the trial. Recruitment was slow, and many pre-screened patients were ineligible or unable to participate. Patients in NYHA classes III and IV often considered the study visits too burdensome, highlighting the need for lower-burden retention strategies in future studies. Recruitment also became more difficult over time as SGLT2 inhibitors were increasingly adopted in routine heart failure care. Consequently, a growing proportion of otherwise potentially eligible patients were already receiving an SGLT2 inhibitor and were excluded. Because two participants withdrew before receiving dapagliflozin and one discontinued at the start of intervention, the analyzed population may represent a selected group able and willing to complete repeated visits and study procedures.

Secondly, it was an open-label trial with fixed sequence so the positive trends seen could be biased due to the placebo effect, performance and detection bias especially for the 6MWT and KCCQ scores. Due the low sample size it is also possible that the results are due to random chance. A random sequence with placebo control would have reduced bias, and a longer pilot trial with more crossovers and participants would have had more power to detect a treatment effect and give more precise effect estimates but would also have increased cost and time commitment for patients while still not being conclusive. Although the AR(1) covariance structure accounted for autocorrelation between repeated measurements, it does not overcome the lack of replication inherent in the single-cycle A–B–A design. With only one treatment–withdrawal sequence per participant, treatment effects cannot be reliably separated from natural variability, time trends, measurement error, or carryover. The model-derived estimates should therefore be interpreted descriptively and cautiously rather than as confirmatory evidence of treatment effect. Future studies with repeated treatment–withdrawal cycles would better allow assessment of reproducibility, within-patient variability, and separation of treatment effects from time-related changes.

Additionally, some analyses – particularly for 6MWT – suffer from missing data points and outlier values that we did not have a prespecified plan for in the statistical analysis plan.

The mixed-effects models used maximum likelihood estimation and included all available observations under a missing-at-random assumption. We considered this assumption plausible because the missing observations were attributable to incomplete questionnaire completion, data-entry error, general fatigue, or a temporary non-cardiac impediment, rather than to the observed outcome values or apparent treatment response. However, because of the small sample size, the missing-at-random assumption could not be formally tested, and even a small number of missing observations may have influenced the estimates. This is particularly relevant for 6MWT, for which the positive trend during the intervention period was partly driven by one outlying value at week 20; disregarding that value substantially reduced the point estimate of effect. The outlier value was verified as correct.

The sensitivity of the 6MWT estimate to a single observation further limits its precision and robustness. Although the 10-week withdrawal period was sufficient for pharmacokinetic elimination and resolution of direct SGLT2 inhibition, the single-cycle design limits interpretation of post-withdrawal changes. Incomplete return to baseline could reflect carryover, longer-lasting physiological adaptation, progression of ATTR-CM, changes in diuretic treatment, or random variation. These explanations cannot be distinguished without repeated treatment-withdrawal cycles or placebo control; withdrawal-period findings should therefore be interpreted descriptively.

## Conclusions

Dapagliflozin was generally well tolerated in patients with ATTR-CM. SBP was lower during intervention than during baseline, without a detected increase in hypotensive symptoms, although this blood pressure effect may be clinically relevant in patients susceptible to hypotension. The estimated differences in NT-proBNP, KCCQ-TSS, and 6MWT distance were imprecise and did not provide conclusive evidence of treatment effects. These findings are exploratory and support evaluation in a larger randomized, placebo-controlled trial.

## Supporting information

S1 File Study protocol. (PDF)

S2 File Statistical analysis plan. (DOCX)

S1 Table Period specific slopes from the prespecified piecewise slope model. (DOCX)

S2 Table Individual data for the three non-completers. (XLSX)
