# Supplementary material for: Using dapagliflozin to reduce symptoms in transthyretin cardiac amyloidosis: An n-of-1 trial series pilot study
Source: PLoS One. 2026 Oct 7;21(10):e0346065. doi: 10.1371/journal.pone.0346065 (PMC13645067; doi:10.1371/journal.pone.0346065)
Supplement: Supplementary file 1 [file pone.0346065.s001.pdf]

CLINICAL STUDY PROTOCOL

A pilot study to evaluate the safety and efficacy of SGLT2 inhibitors in patients with cardiac amyloidosis

ULRICA-PILOT

Study code:	ULRICA-PILOT
EudraCT number:	2021-003674-32
Version number:	1.1
Date:	2022-12-05

Sponsor and Coordinating Investigator:	Krister Lindmark

Table Of Contents

Signature page ... 5
Contact information ... 7
List of used acronyms and abbreviations ... 9
1. Synopsis ... 11
2. Background and rationale ... 14
3. Benefit-risk evaluation ... 16
4. Study objectives ... 17
4.1. Primary objective ... 17
4.2. Secondary objective(s) ... 17
4.3. Primary endpoint ... 18
4.4. Secondary endpoint ... 18
5. Study design and procedures ... 18
5.1. Overall study design ... 18
5.2. Procedures and flow chart ... 18
5.3. Biological sampling procedures ... 20
5.4. End of Study ... 21
6. Subject selection ... 21
6.1. Inclusion criteria ... 21
6.2. Exclusion criteria ... 22
6.3. Screening visit ... 22
6.4. Withdrawal criteria ... 23
7. Study treatments ... 23
7.1. Description of investigational product ... 23
7.2. Dose and administration ... 23
7.3. Packaging, labeling, and handling of investigational products(s) ... 24
7.4. Drug accountability and treatment compliance ... 24
7.5. Concomitant medications ... 24
7.6. Treatment after study end ... 24
8. Methods for measurement of endpoints for clinical efficacy and safety ... 25
8.1. Methods for measurement of endpoints for clinical efficacy ... 25
8.1.1. Primary endpoint ... 25

8.1.2. Secondary endpoints ... 25
8.2. Methods for measurement of endpoints for clinical safety ... 25
9. Handling of Adverse Events ... 26
9.1. Definitions ... 26
9.1.1. Adverse Event (AE) ... 26
9.1.2. Serious Adverse Event (SAE) ... 26
9.1.3. Suspected Unexpected Serious Adverse Reaction (SUSAR) ... 26
9.2. Assessment of Adverse Events ... 26
9.2.1. Assessment of causal relationship ... 26
9.2.2. Assessment of intensity ... 27
9.2.3. Assessment of seriousness ... 27
9.3. Reporting and registration of Adverse Events ... 28
9.3.1. Reporting of Adverse Events (AE) ... 28
9.3.2. Reporting of Serious Adverse Events (SAE) ... 28
9.3.3. Reporting of Suspected Unexpected Serious Adverse Reactions (SUSAR) ... 28
9.4. Follow-up of Adverse Events ... 29
9.5. Annual Safety Report (Development Safety Update Report, DSUR) ... 29
9.6. Procedures in case of emergencies, overdose or pregnancy ... 29
9.7. Reference Safety Information ... 29
10. Statistics ... 29
10.1. Model the difference in outcome between periods ... 29
10.2. Model the difference and the change in outcome over time between periods ... 30
10.3. Statistical analysis population ... 30
11. Quality Control and Quality Assurance ... 30
11.1. Quality Assurance and Sponsor oversight ... 30
11.2. Monitoring ... 30
11.3. Source data ... 31
11.4. Deviations or serious breaches ... 31
11.5. Inspections ... 31
12. Ethics ... 31
12.1. Compliance to the protocol, GCP and regulations ... 31
12.2. Ethical review of the study ... 31
12.3. Procedure for obtaining informed consent ... 32

12.4. Data protection 32
12.5. Insurances 32
13. Substantial changes to the study 33
14. Collection, handling, and archiving data 33
14.1. Case Report Form 33
15. Notification of study completion, reporting, and publication 34
16. References 34
17. Attachments 36

Signature page

Sponsor / Coordinating Investigator

I am responsible for ensuring that this protocol includes all essential information to be able to conduct this study. I will submit the protocol and all other important study-related information to the responsible investigator(s) so that they can conduct the study correctly. I am aware that it is my responsibility to hold the staff members who work with this study informed and trained.

By signing my name below, I agree to conduct the study in compliance with this protocol, the Declaration of Helsinki, ICH GCP (Good Clinical Practice) guidelines and the current national and international regulations governing the conduct of this clinical trial.

I will submit this protocol and all other important study-related information to the staff members and investigators who participate in this study, so that they can conduct the study correctly. I am aware of my responsibility to continuously keep the staff members and investigators who work with this study informed and trained.

I am aware that quality control of this study will be performed in the form of monitoring, audit, and possibly inspection.

____

Sponsor / Coordinating Investigator's signature   Date

____

Printed name

Principal Investigator

I have read this protocol and agree that it includes all essential information to be able to conduct the study. By signing my name below, I agree to conduct the study in compliance with this protocol, the Declaration of Helsinki, ICH GCP (Good Clinical Practice) guidelines and the current national and international regulations governing the conduct of this clinical trial.

I will submit this protocol and all other important study-related information to the staff members and investigators who participate in this study, so that they can conduct the study correctly. I am aware of my responsibility to continuously keep the staff members and investigators who work with this study informed and trained.

I am aware that quality control of this study will be performed in the form of monitoring, audit, and possibly inspection.

____

Principal Investigator’s signature Date

____

Printed name

Contact information

**Role**	
Sponsor/Coordinating Investigator	Krister Lindmark, MD PhD<br><br>Department of Public Health and Clinical Medicine<br>Umeå University<br><br>070-288 82 85<br><br>Krister.lindmark@umu.se
Principal Investigator Umeå	Björn Pilebro, MD PhD<br><br>Hjärtcentrum<br><br>Bjorn.pilebro@regionvasterbotten.se<br><br>076 801 69 97
Sub-investigator	Erik Håkansson, MD<br><br>Hjärtcentrum<br><br>Erik.hakansson@regionvasterbotten.se
Sub-Investigator	Jonas Wixner, MD, PhD<br><br>Medicincentrum<br><br>Jonas.wixner@regionvasterbotten.se<br><br>070 645 56 24
Sub-Investigator<br><br>Intissar Annan	Intissar Anan, MD, PhD<br><br>Medicincentrum<br><br>Intissar.anan@umu.se
Sub-Investigator	Mattias Brunström, MD PhD<br><br>Hjärtcentrum<br><br>mattias.brunstrom@umu.se

**Role**	
Clinical monitoring organization	Kliniskt forskningscentrum (KFC)<br>Region Västerbotten

List of used acronyms and abbreviations

Abbreviation	Term/Explanation
6MWT	6 minutes walking test
AE	Adverse Event = any untoward medical occurrence
AL-amyloidosis	Amyloidosis light chain
AR	Adverse Reaction = adverse event, that is each unfavorable and unexpected reaction to a study treatment, regardless of dose
ATTR-CM	Amyloid Transthyretin cardiomyopathy
ATTRv	Amyloid Transthyretin variant – a hereditary form
ATTRwt	Amyloid Transthyretin wild type – an acquired form
AV-block	Atrial Ventricular block
CIOMS-form	Council for International Organisations of Medical Sciences form
CRF	Case Report Form
CTFG guidelines	Clinical Trials Facilitation and Coordination Group
DPD	3,3-diphosphono-1,2-propanodicarboxylic acid (DPD) for detection of cardiac ATTR deposits
DSUR	Development Safety Update Report = annual safety report
EPM	Etikprövningsmyndigheten (English: Swedish Ethical Review Authority)
ESC	European Society of Cardiology
GCP	Good Clinical Practice
GLS	Global Longitudinal Strain
HF	Heart Failure
hs-troponin	High sensitivity troponin
ICH	International Council for Harmonization
ITT	Intention-to-treat = including all data from all subjects who have participated in the study
KCCQ	Kansas City Cardiomyopathy Questionnaire

LVFS	Läkemedelsverkets författningssamling (English: Swedish Medical Products Agency's statutes)
NTproBNP	N-terminal B-type Natriuretic Peptide
NYHA	New York Heart Association – classification system for heart failure
PP	Per Protocol analysis = including only data from subjects who have completed the study completely in accordance with the protocol, with no deviations from the protocol
RCT	Randomised Controlled Trial
SAE	Serious Adverse Event = serious untoward medical occurrence
SGLT2	Sodium-Glucose cotransporter type 2
SPC or SmPC	Summary of Product Characteristics
SUSAR	Suspected Unexpected Serious Adverse Reaction
TTE	Transthoracic echocardiography

1. Synopsis

EudraCT number:	2021-003674-32
Title:	A pilot study to evaluate the safety and efficacy of SGLT2 inhibitors in patients with cardiac amyloidosis
Study code:	ULRICA-PILOT
Short background/ Rationale/Aim:	Transthyretin amyloid (ATTR) amyloidosis is a fatal and disabling systemic disease. Patients with cardiac involvement generally have severe symptoms and traditional heart failure (HF) treatments are ineffective and often lead to intolerable side effects. SGLT2 -inhibition is a new mechanism for treatment of HF that theoretically could be beneficial for patients with cardiac ATTR amyloidosis.<br><br>The purpose of the exploratory study is to obtain baseline knowledge on tolerability and efficacy of SGLT2 inhibitors in cardiac amyloidosis as a basis for future larger RCTs.
Study objectives:	Primary objective:<br><br>• Assess impact of dapagliflozin on NT-proBNP levels in patients with optimally treated cardiac ATTR amyloidosis<br><br>Secondary objectives:<br><br>• determine whether dapagliflozin reduces patient-reported HF symptoms in patients with optimally treated cardiac ATTR amyloidosis.<br>• assess whether dapagliflozin improves walking distance at 6MWT in patients with cardiac ATTR amyloidosis
Study design:	Open label phase II study. Single subject experimental design/N-of-1 trial series. ABA-design (baseline, intervention, withdrawal).
Study population:	Patients treated for cardiac ATTR amyloidosis
Number of subjects:	10 completing the trial
Inclusion criteria:	1 Established diagnosis of ATTRv or ATTRwt amyloidosis verified by either:<br>A Tissue biopsy with ATTR amyloid deposits

	OR<br>B DPD scintigraphy with Perugini scale grade II or III cardiac uptake and absence of monoclonal bands in serum and urine electrophoresis and normal free light chain assay ruling out AL amyloidosis.<br><br>2 Clinical diagnosis of HF according to ESC criteria for diagnosing HF with symptoms of heart failure and a left ventricular ejection fraction of <50%, or echocardiographic signs of diastolic dysfunction according to ESC criteria.<br><br>3 NYHA-class II-IV<br><br>4 Intraventricular septum thickness >12 mm<br><br>5 DPD scintigraphy uptake in the myocardium Perugini scale grade II or III<br><br>6 Optimal treated and stable HF and ATTR amyloidosis for at least four weeks, according to the treating physician (see section 7.5 for further details).<br><br>7 Genetic testing is performed on all patients to determine diagnosis of ATTRwt or ATTRv<br><br>8 Age ≥ 18<br><br>9 TTE performed within 12 months of screening visit.<br><br>10 Women of Childbearing Capacity (WOCBC) must comply to use of highly effective contraception methods during the trial. Acceptable methods according to CTFG guidelines are combined hormonal contraception (oral, dermal, intravaginal), progesterone-only hormonal contraception associated with inhibition of ovulation (oral, injectable, implantable), an intrauterine device, an intrauterine hormone-releasing system or by refraining from heterosexual intercourse during the entire period of risk.
Exclusion criteria:	1. Expected survival < 9 months<br><br>2. Estimated glomerular filtration rate <25 ml/ min<br><br>3. Karnofsky performance status <60%

	4. Diabetes mellitus treated with insulin or sulfonylureas<br>5. Acute cardiac hospitalization or procedure within 4 weeks (HF hospitalization, myocardial infarction, pacemaker implantation, cardioversion, coronary angioplasty)<br>6. Participation in other clinical trial not approved for co-enrollment<br>7. Inability or unwillingness to comply with the study requirements<br>8. Current SGLT2-inhibitor use or previous discontinuation due to side effects<br>9. History of ketoacidosis<br>10. Systolic blood pressure <90 mmHg<br>11. Uncontrolled hypertension<br>12. Severe hepatic impairment, unstable or rapidly progressing hepatic disease at the time of randomization as judged by the investigator.
Investigational product(s), dosage, administration:	Dapagliflozin 10 mg daily
Study endpoints:	Primary endpoints:<br>• Change in level or slope of NTproBNP during the treatment period compared to the non-treatment periods<br>Secondary endpoint(s):<br>• Change in Kansas City Cardiomyopathy Questionnaire total symptom score during the treatment period compared to the non-treatment periods<br>• Change in 6-minute walking distance during the treatment period compared to the non-treatment periods<br>Safety endpoints:<br>• Blood pressure

	• Diuretic intake<br>• Any patient-reported side effect during treatment period with dapagliflozin
Study period:	Q1 2023 – Q1 2024

2. Background and rationale

ATTR amyloidosis is a systemic amyloidosis caused by misfolded transthyretin molecules that forms cross-β-sheet-rich amyloid fibrils in several tissues (Westermark et al. 1990). The disease exists in a hereditary (ATTRv) and an acquired (ATTRwt) form (Westermark et al. 2003). If the amyloid fibrils accumulate in the myocardium it can lead to amyloid cardiomyopathy (ATTR-CM) characterized by dyspnea, fatigue, orthostatic hypotension, and conduction disturbances including AV-block and atrial fibrillation. Median survival after diagnosis of ATTR-CM in untreated patients has been reported to be 2.5 years for ATTRv and 3.6 years for ATTRwt (Grogan et al. 2016).

The disease has caught interest in recent years due to two major developments. The first is the development of non-invasive diagnosis of ATTR-CM by technetium diphosphonate bone scintigraphy, e.g., 3,3-diphosphono-1,2-propanodicarboxylic acid (DPD) for the detection of cardiac ATTR deposits (Perugini et al. 2005). The second development is emerging treatments for ATTR-CM. Tafamidis has shown a reduction in mortality in patients in relatively early stages of cardiac ATTRv amyloidosis (Maurer et al. 2018). RNA-interference for treatment of ATTRv was listed by Science as one of the top-ten scientific breakthroughs in 2018 and showed improvements of multiple clinical manifestations of ATTRv in a study with professor Ole Suhr of Umeå University as senior last author (Adams et al. 2018). There are also phase-I clinical trials in the upstart phase using the DNA-technique CRISPR/Cas-9. Our centre will be a participating site in this study.

The treatments that have been introduced or are in development are directed towards the early stages of the disease and modifying disease progression. The mortality curves for tafamidis vs placebo started to diverge after 18 months of treatment and efficacy was reduced in patients with more advanced heart failure symptoms (Maurer et al. 2018). RNA- and DNA-based therapies are so far also only directed towards the hereditary ATTRv, not ATTRwt. Recent data from our group suggest that the prevalence of ATTRwt is more common than previously thought (Lindmark et al. 2021). With the accumulation of amyloid fibrils in the myocardium the elasticity of the myocardium is reduced and myocardial hypertrophy develops (Ruberg et al. 2019). Emphasis has traditionally been on diastolic dysfunction leading to heart failure with preserved ejection fraction (HFpEF) but recent data suggests that heart failure with reduced ejection fraction (HFrEF) is also common (Lindmark et al. 2021). With the reduced ventricular capacitance maintaining euvolumia is challenging. The patients are sensitive to over aggressive diuresis and vasodilation causing symptomatic hypotension, but also to volume overload leading to circulatory congestion. Traditional heart failure (HF) medications, i.e. angiotensin converting enzyme inhibitors, beta blockers, mineral

corticoid receptor antagonists and angiotensin receptor neprilysin inhibitors are usually not well tolerated and may even be harmful according to observational studies (Aus dem Siepen et al. 2017).

Sodium–glucose cotransporter 2 (SGLT2)-inhibitors are a class of drugs used for diabetes type 2 which lowers blood sugar by reducing the reuptake of glucose from the proximal convoluted tubule of the nephron to induce a glucose-mediated osmotic diuresis and natriuresis. It has been shown that the drug class reduces the risk for hospitalization for HF in patients with type 2 diabetes and has in two landmark clinical trials showed effects on mortality and HF hospitalization in patients with HF with- and without diabetes (McMurray et al. 2019, Packer et al. 2020). The exact mechanisms responsible for the cardioprotective effects and the benefits seen in HF are not completely clear. Several distinct mechanisms have been proposed and could arguably be beneficial for patients with amyloid cardiomyopathy, regardless of ejection fraction: improved loading conditions due to osmotic diuresis and natriuresis, lowering of arterial stiffness, change of myocardial metabolism with usage of more ketone bodies, reversing cardiac fibroses by inhibition of the sodium/hydrogen exchanger in the myocardium, improving diastolic function as well as decreasing peripheral vascular resistance (Bonner et al. 2015, Ferrannini, Mark and Mayoux 2016, Kang et al. 2020, Solini et al. 2017, Verma et al. 2018).

There are many causes for HF and the most common etiologies are coronary heart disease and hypertension. Clinical trials for HF usually do not consider the etiology but are instead directed towards the phenotypes according to ejection fraction (HFrEF, HFmrEF and HFpEF). Cardiac ATTR amyloidosis has several traits that distinguishes this group from other types of HF and regular HF treatments are usually not well tolerated (Aus dem Siepen et al. 2017). To date there is no treatment that has proved to alleviate symptoms, prolong survival, or reduce hospitalizations in the later stages of cardiac ATTR amyloidosis. There is a need for treatments directed to the highly symptomatic patients with cardiac ATTR amyloidosis. To our knowledge, there are hitherto no studies on SGLT2-inhibitors in patients with ATTR amyloidosis.

The hereditary form, ATTRv is common in Västerbotten county due to a high prevalence of the mutation V30M. In the areas of Skellefteå and Lycksele the disease prevalence has been estimated to be 1000/million inhabitants and the total prevalence for Sweden 26/million inhabitants (Suhr et al. 2017). Several research projects have been conducted by Umeå University amyloidosis centre. Due to the high research activity in the field the population with ATTRv is thoroughly mapped in our centre. Globally, the wild type, ATTRwt is more common but prevalence numbers are scarce due to a slow spread of the awareness of the disease and diagnostic capabilities. Our group showed that approximately 20% of patients with HF and increased myocardial thickness has ATTRwt (Lindmark et al. 2021). From a clinical sample of this patient group at Umeå University hospital (n=27), NTproBNP levels had a mean value of 2800 ng/L and correlate to symptoms of HF (unpublished data). Due to the high prevalence of ATTRv, the awareness and mapping of patients with ATTRwt as well as our amyloidosis research centre.

3. Benefit-risk evaluation

SGLT2-inhibitors are established as treatment for type 2 diabetes and heart failure and have been shown to reduce the risk of hospitalization due to heart failure, both for patients with and without type 2 diabetes. The exact mechanism of the cardioprotective effect has not been established.
The effect of SGLT2-inhibitors is untested for the patient group in this study. Patients with ATTR amyloidosis with cardiac effects generally have severe symptoms and traditional heart failure treatments are ineffective and often lead to unacceptable side effects for this patient group with severe drops in blood pressure. There is a need for effective treatment for this patient group and this study could lead to the first accepted treatment for this patient group.

The side effects seen with SGLT2-inhibitors are in most cases mild and easy to manage. Possible side effects of SGLT2-inhibitors are mainly urogenital infections that can occur as the mechanism of action causes glucose to be excreted in the urine. These are usually uncomplicated and easy to treat. For the same reason, patients may experience increased urine output. Patients who have other diabetes treatment may experience changes in blood sugar levels that should be monitored closely when starting treatment. More serious side effects are rare but that can occur include diabetic ketoacidosis in patients who have very long-term type 2 diabetes and so-called Fournier's gangrene which is a rare but dreaded complication. In the studies that form the basis of approval for SGLT2-inhibitors with more than 4,000 treated patients, there was not a single case of Fournier's gangrene in the treatment group (McMurray et al 2019)

Reactions from taking blood samples may occur, e.g. fainting, redness, pain, bruising, bleeding or infection. The patient may also experience minor discomfort from the ultrasound examination of the heart since the patient needs to lie still in the same position during the examination.

Since this patient group are sensitive to volume depletion and may react with orthostatic hypotension there is a theoretical risk that some of the patients may experience symptoms from this on active study medication. All patients will be instructed to contact the study site if this occurs. This side effect was not a problem in the overall HF-trials with SGLT2-inhibitors (McMurray et al 2019, Packer et al 2020) but may prove to be important in this group.

The clinical experience with SGLT2-inhibitors in HF is that some patients may need to reduce the dose of loop diuretics during treatment. Loop diuretics are considered as a symptomatic treatment and does not improve the prognosis. In HF guidelines it is suggested to try to lower the dose of loop diuretics if possible (Ponikowski et al 2016). Therefore, this should not be a significant problem and the patients will receive instructions from the study nurse on how to monitor the body weight and discuss diuretic intake with the study site.

There have been post-marketing reports of ketoacidosis, including diabetic ketoacidosis, in patients with type 2 diabetes mellitus taking dapagliflozin and other SGLT2 inhibitors. Diabetic ketoacidosis is considered a rare (in ≥1/10000 to <1/1000 patients) adverse drug reaction for dapagliflozin in patients with type 2 diabetes mellitus. Patients treated with

dapagliflozin who present with signs and symptoms consistent with ketoacidosis, including nausea, vomiting, abdominal pain, malaise, and shortness of breath, should be assessed for ketoacidosis, even if blood glucose levels are below 14 mmol/L. If ketoacidosis is suspected, interruption of dapagliflozin treatment should be considered, and the patient should be evaluated promptly. Predisposing factors to ketoacidosis include a low beta-cell function reserve resulting from pancreatic disorders (eg, type 1 diabetes, history of pancreatitis, or pancreatic surgery), insulin dose reduction, reduced caloric intake, or increased insulin requirements caused by infections, illness or surgery and alcohol abuse. Dapagliflozin should be used with caution in patients in these circumstances. Dapagliflozin is currently not indicated for the treatment of patients with type-1 diabetes mellitus; these patients are excluded from this study. Hypoglycaemia is not common with SGLT2-inhibitors but patients who already have diabetes will be instructed to monitor their plasma glucose levels more carefully in the beginning of each study period to make sure that the levels do not change significantly.

An increase in amputations, mostly affecting toes, was observed in a clinical trial (Neal et al 2017) with another SGLT2 inhibitor. There is no indication from the clinical development programme that dapagliflozin is associated with an increased risk of amputation. As a precaution, lower limb examination will be included in the physical examination.

Overall, it is judged that potential great benefits outweigh the small risks.

4. Study objectives

Transthyretin amyloid (ATTR) amyloidosis is a fatal and disabling systemic disease(Suhr et al. 2017). Patients with cardiac involvement generally have severe symptoms and traditional HF treatments are ineffective and often lead to intolerable side effects (Aus dem Siepen et al. 2017). SGLT2 -inhibition is a new mechanism for treatment of HF(Packer et al. 2020, McMurray et al. 2019) that theoretically could be beneficial for patients with cardiac ATTR amyloidosis. The purpose of the study is to obtain basic knowledge on tolerability and efficacy of SGLT2 inihibitor dapagliflozin in patients with cardiac amyloidosis as a basis for future larger RCTs.

4.1. Primary objective

The primary objective of this study is to assess impact of dapagliflozin on NT-proBNP levels in patients with optimally treated cardiac ATTR amyloidosis to provide data as a basis for future RCTs on the subject

4.2. Secondary objective(s)

To determine whether dapagliflozin reduces patient-reported HF symptoms in patients with optimally treated cardiac ATTR amyloidosis.

To assess whether dapagliflozin is improving walking distance during a 6MWT in patients with cardiac ATTR amyloidosis

4.3. Primary endpoint

The primary endpoints are:

- Change in level or slope of NTproBNP during the treatment period compared to the non-treatment periods

4.4. Secondary endpoint

1. Change in Kansas City Cardiomyopathy Questionnaire total symptom score during the treatment period compared to the non-treatment periods
2. Change in 6-minute walking distance during the treatment period compared to the non-treatment periods.

Safety endpoints (variables)

- Blood pressure
- Diuretic intake
- Any patient-reported side effect during treatment period with dapagliflozin vs no treatment period.

5. Study design and procedures

5.1. Overall study design

Open label phase II study. Single subject experimental design/N-of-1 trial series. ABA-design (baseline, intervention, withdrawal) investigating the impact of dapagliflozin 10 mg daily on NTproBNP levels, 6MWT and patient reported HF symptoms in patients with optimally treated cardiac ATTR amyloidosis. Since there is no blinding or randomisation, this is mainly an exploratory study to provide data for possible future RCTs on the subject

5.2. Procedures and flow chart

Patients will be recruited from the patient population treated for cardiac ATTR amyloidosis at Umeå university hospital. Patients with a prevalent diagnosis will be asked to participate in the study as well as newly diagnosed patients. All patients with known cardiac ATTR amyloidosis in the county are treated at the amyloidosis centre. Patient visits and examinations will be conducted at the Umeå university hospital. A total of 10 patients, completing the trial, will be recruited for the study during an expected period of 12 months.

A screening visit will be performed up to 4 weeks prior to visit A1. Informed consent will be documented in CRF and patient medical records after written and spoken information about

study procedures. Subject eligibility (that subjects fulfill all inclusion criteria and do not meet any exclusion criteria) is established before inclusion. Patients' medical history will be reviewed, and information entered in CRF. Vital signs and physical examination will be recorded with recording of blood pressure, heart rate, respiratory rate, oxygen saturation, auscultation of heart and lungs, examination of lower extremities, signs of congestion and other examination deemed necessary by the investigator.

Each subject will have 30 weeks of study participation, starting with a baseline period of 8 weeks without treatment with dapagliflozin with 5 study visits every second week (A1-A5). After visit A5, patients will start dapagliflozin treatment for 12 weeks with 6 study visits every second week (B1-B6), followed by a 10 week wash-out period, with 5 study visits every second week (A6-A10). Visits will be scheduled as close to every 14 days as possible, but a difference in 4 days earlier or later will be accepted. See table 1.

At every study visit:

- Laboratory collection: Chemistry including: NT-proBNP, creatinine, hematology, sodium, potassium, hs-troponin T. In diabetic patients, fasting B-glc and HbA1C will be analysed.
- Anamnesis: Side effects, fall, other adverse events, reported intake of diuretics
- Vital signs: Blood pressure, heart rate, weight, respiratory rate, oxygen saturation and body temperature.

3 times per period (i.e. A1, A3, A5, B1, B3, B5, A6, A8, A10):

1. 6 minutes walking test (6MWT)
2. KCCQ

1 time per period (i.e. A5, B6, A10):

3. Transthoracic echocardiogram

Week	Visit	Vital signs	Physical	Chemistry	6MWT	KCCQ	TTE
**Screening visit**	**A0**	x	x	x			
**V0**	**A1**	x	x	x	x	x	
**V2**	**A2**	x		x			
**V4**	**A3**	x	x	x	x	x	
**V6**	**A4**	x		x			
**V8**	**A5**	x	x	x	x	x	x
**v10**	**B1**	x		x			
**v12**	**B2**	x	x	x	x	x	
**v14**	**B3**	x		x			
**v16**	**B4**	x	x	x	x	x	
**v18**	**B5**	x		x			
**v20**	**B6**	x	x	x	x	x	x
**v22**	**A6**	x	x	x	x	x	
**v24**	**A7**	x		x			
**v26**	**A8**	x	x	x	x	x	
**v28**	**A9**	x		x			
**V30**	**A10** Study completion	x	x	x	x	x	x

5.3. Biological sampling procedures

Blood samples will be collected at every visit as a part of normal clinical routine.

Biobank act does not apply since all samples taken in this study are part of clinical routine and will be analyzed within six months of the date of sampling and destroyed immediately after analysis.

5.4. End of Study

The study ends when the last subject has completed the last follow-up. The study may be prematurely terminated if it appears that the treatment involved a large number of serious adverse events (SAE) or if recruitment of subjects cannot be met within reasonable time limits. If the study is prematurely terminated or suspended, the investigator should immediately inform the subjects about this and ensure appropriate treatment and follow-up. The regulatory authority should be informed as soon as possible, but no later than within 15 days.

Decisions on premature termination are taken by the sponsor.

6. Subject selection

6.1. Inclusion criteria

To be included in the study, subjects must meet the following criteria:

1. Established diagnosis of ATTRv or ATTRwt amyloidosis verified by either:
   1. Tissue biopsy with ATTR amyloid deposits

OR

   2. DPD scintigraphic uptake in the myocardium Perugi scale grade II or III and absence of monoclonal bands in serum and urine electrophoresis and normal free light chain assay ruling out AL amyloidosis.

2. Clinical diagnosis of HF according to ESC criteria for diagnosing HF with symptoms of heart failure and a left ventricular ejection fraction of <50%, or echocardiographic signs of diastolic dysfunction according to ESC criteria.

3. NYHA-class II-IV

4. Intraventricular septum thickness >12 mm

5. DPD scintigraphic uptake in the myocardium Perugi scale grade II or III

6. Optimally treated HF and ATTR amyloidosis for at least four weeks, according to the treating physician.

7. Genetic testing is performed on all patients to determine diagnosis of ATTRwt or ATTRv

8. Age ≥ 18

9. The subject has given written consent to participate in the study.

10. Women of Childbearing Capacity (WOCBC) must comply to use of highly effective contraception methods during the trial. Acceptable methods according to CTFG guidelines are combined hormonal contraception (oral, dermal, intravaginal), progesterone-only hormonal contraception associated with inhibition of ovulation (oral, injectable, implantable), an intrauterine device, an intrauterine hormone-releasing system or by refraining from heterosexual intercourse during the entire period of risk.
11. Echocardiogram performed within 1 year of screening visit

6.2. Exclusion criteria

Subjects must not be included in the study if any of the following criteria are met:

1. Expected survival < 9 months
2. Estimated glomerular filtration rate <25 ml/ min
3. Karnofsky performance status <60%
4. Diabetes mellitus treated with insulin or sulfonylureas
5. Acute cardiac event withing 4 weeks of screening visit (HF hospitalization, acute myocardial infarction, cardioversion, pacemaker implantation, coronary angioplasty)
6. Participation in other clinical trial not approved for co-enrollment
7. Inability or unwillingness to comply with the study requirements
8. Current SGLT2-inhibitor use or previous discontinuation due to side effects
9. History of ketoacidosis
10. Systolic blood pressure <90 mmHg
11. Uncontrolled hypertension
12. Severe hepatic impairment, unstable or rapidly progressing hepatic disease at the time of randomization as judged by the investigator.

6.3. Screening visit

Informed consent will be documented in CRF and patient medical records after written and spoken information about study procedures. Subject eligibility (that subjects fulfill all inclusion criteria and do not meet any exclusion criteria) is established before inclusion. Patients'

medical history will be reviewed, and information entered in CRF. Vital signs and physical examination will be recorded.

6.4. Withdrawal criteria

Subjects can discontinue their participation in the study at any time without any consequence to his/her continued treatment. The investigator/sponsor can at any time terminate the study for a subject due to, e.g., unacceptable adverse events/adverse reactions or because the subject does not follow procedures in the study protocol. If the subject discontinues the study, follow-up of this subject will be performed according to the clinic's routine. If the test subject has a urinary tract infection during the active treatment period, the study treatment should be temporarily paused. After the symptoms of the infection has subsided the investigator will together with the test subject decide if the active treatment should be recommenced. If the systolic blood pressure of the test subject drops below 90 mmHg together with increased symptoms of orthostatic hypotension the treatment should be discontinued. Test subjects with diabetes who withdraw from the study after exposure to the study medication will receive further testing for B-glucose as needed at the discretion of the investigator and type 2 diabetes mellitus treatment may be adjusted at the discretion of the investigator or diabetes health care provider.

7. Study treatments

7.1. Description of investigational product

Each subject will start with a baseline period of 8 weeks without treatment with the investigational product (Dapagliflozin). The baseline period is followed by 12 weeks of treatment with Dapagliflozin 10 mg daily, followed by a 10-week wash-out period. Dose modifications are not permitted.

7.2. Dose and administration

The dose that has been chosen, Dapagliflozin 10 mg once daily is the same dose that was chosen for the landmark clinical trial with Dapagliflozin in heart failure, DAPA-HF.

Table 2 Investigational product

	Investigational product
Investigational product	Dapagliflozin 10 mg
Dose formulation	Film coated tablets
Unit dose strength	10 mg
Dose Level	10 mg once daily

Route of administration	Per oral

7.3. Packaging, labeling, and handling of investigational products(s)

The investigational product is an approved medicinal product (Forxiga®) in Sweden. There will be no additional labelling for clinical trial of the investigational products. The investigational product is prescribed by the investigators and the subjects receive it at a local pharmacy.

7.4. Drug accountability and treatment compliance

Compliance will be recorded by patients' self-reported information. Compliance is documented in the Case Report Forms (CRFs).

7.5. Concomitant medications

Medications that are considered necessary for the safety and well-being of the subject can be given at the discretion of the investigator, unless otherwise specified as an exclusion criteria. Concomitant medications should be reported in the CRF.

Diabetes medications at baseline and during the study will be recorded in the CRF. Patients with type 2 diabetes mellitus at study start will continue their type 2 diabetes mellitus treatment. SGLT2-inhibitors are prohibited as per exclusion criteria. Patients treated with insulin or insulin secretagogues have a higher risk of experiencing hypoglycaemic events compared with those treated with other anti-diabetic agents. If needed, type 2 diabetes mellitus treatments may be adjusted at the discretion of the Investigator or diabetes health care provider.

Cardiac ATTR amyloidosis should be optimally treated since at least four weeks prior to inclusion according to the treating physician in accordance with ESC position paper (Garcia-Pavia et. al. 2021). Patients should receive diuretics as needed and have been considered for Tafamidis and Patisiran.

7.6. Treatment after study end

After study end there will be no additional intervention. Patients will receive standard of care according to national guidelines for Cardiac ATTR Amyloidosis.

8. Methods for measurement of endpoints for clinical efficacy and safety

8.1. Methods for measurement of endpoints for clinical efficacy

8.1.1. Primary endpoint

The primary endpoint is ng/L change in serum levels of NTproBNP during the treatment period compared to the non-treatment periods and slope of change of during the treatment period compared to the non-treatment periods. NTproBNP is released from cardiomyocytes in response to changes in intracardiac pressures and is widely used as a marker for severity of HF. Measurements of NTproBNP will be made at the local laboratory at Umeå university hospital continuously during the study.

8.1.2. Secondary endpoints

Change in Kansas City Cardiomyopathy Questionnaire (KCCQ) total symptom score during the treatment period compared to the non-treatment periods. The KCCQ is a self-administered 23 item instrument, measuring: physical functioning, symptoms, social functioning, self-efficacy and knowledge and quality of life. Scores are transformed to a range from 0-100 in which higher score reflects better outcome.

Change in 6-minute walking distance measured in meters during the treatment period compared to the non-treatment periods. The 6-minute walk test is validated instrument in HF to assess the functional capacity where the test subject walks as far as possible during six minutes at a measured test track.

Change in percentage level of global longitudinal strain measured by echocardiography at the end of the treatment period with dapagliflozin vs at the end of the no treatment period. GLS is an echocardiographic parameter that measures longitudinal shortening expressed as a percentage change in length proportional to baseline length.

8.2. Methods for measurement of endpoints for clinical safety

The safety endpoint includes clinical evaluation day 0, 14, 28, 56, 84, 112, 140, 154, 182, 210.

Patient-reported orthostatic hypotension at the end of the treatment period with dapagliflozin vs at the end of the no treatment period. This will be assessed by clinical evaluation and direct questions to the patient.

Patient reported syncope at the end of the treatment period with dapagliflozin vs at the end of the no treatment period. This will be assessed by clinical evaluation and direct questions to the patient.

Rise of Creatinine over 30% at the end of the treatment period with dapagliflozin vs at the end of the no treatment period. This will be assessed by blood samples taken at each study visit and measured continuously at the local lab during the study.

Any hospitalization during treatment period with dapagliflozin vs during the no treatment period. This will be assessed by clinical evaluation and direct questions to the patient.

Any patient-reported side effect during treatment period with dapagliflozin vs during the no treatment period. This will be assessed by clinical evaluation and direct questions to the patient.

The local routine, and measurement methods available, will be used to evaluate heart rate (per minute), respiration frequency (per minutes) blood pressure (Systolic blood pressure/diastolic blood pressure) and saturation (the oxygen supply l/min will be noted).

9. Handling of Adverse Events

9.1. Definitions

9.1.1. Adverse Event (AE)

Adverse Event (AE): Any untoward medical occurrence in a clinical investigation subject administered a medicinal product and, which does not necessarily have a causal relationship with the treatment, can be an unfavorable and unintended sign (including an abnormal laboratory discovery), symptom or disease temporally associated with the use of the medicinal (investigational) product, whether or not related to the medicinal (investigational) product.

9.1.2. Serious Adverse Event (SAE)

Serious adverse event (SAE): Any untoward medical occurrence that at any dose:

- results in death
- is life-threatening
- requires inpatient hospitalization or prolongation of existing hospitalization
- results in persistent or significant disability or incapacity
- results in a congenital anomaly/malformation

Medical and scientific assessment will be made to determine if an event is “serious” and whether it would prompt reporting in other situations, for example important medical events that may not be directly life-threatening or result in death or hospitalization but may compromise the study subject or may require intervention to prevent one of the other results set forth in the definitions above. These should also normally be considered as SAEs.

9.1.3. Suspected Unexpected Serious Adverse Reaction (SUSAR)

SUSAR: A reaction/event that is unexpected, serious, and suspected to be caused by the treatment, i.e. adverse events that are not included in SPC.

9.2. Assessment of Adverse Events

9.2.1. Assessment of causal relationship

The investigator is responsible for determining whether there is a causal relationship between the AE/SAE and use of the investigational product.

Those AEs which are suspected of having a relationship to the investigational product will be followed up until the subject has recovered or is well taken care of and on their way to good recovery (see also section 9.4, Follow-up of Adverse Events).

All AE will be categorized either as likely related, possibly related, or not related, in accordance with the definitions below:

Likely related: Clinical event, including abnormal results from laboratory analyses, occurring within a reasonable time after administration of the intervention/investigational product. It is unlikely that the event can be attributed to underlying disease or other medications but is most likely caused by the investigational product and its emergence is reasonable in relationship with use of the investigational product.

Possibly related: Clinical event, including abnormal results from laboratory analyses, occurring within a reasonable time after administration of the intervention/investigational product. The event could be explained by the investigational product and its emergence is reasonable in relationship with use of the investigational product, but there is insufficient information to determine the relationship. The event could be explained by an underlying disease or other medications.

Unlikely related: The event is unlikely related to the intervention/investigational product and can be explained by other medications or underlying disease.

Not related: Clinical event, including abnormal results from laboratory analyses, that is not reasonably related to the use of the intervention/investigational product.

9.2.2. Assessment of intensity

Each adverse event shall be classified by an investigator as mild, moderate or severe.

Mild: The adverse event is relatively tolerable and transient in nature but does not affect the subject's normal life.

Moderate: The adverse event causes deterioration of function but does not affect health. The event can be sufficiently unpleasant and interferes with normal activities but does not completely obstruct them.

Severe: The adverse event causes deterioration of function or work ability or poses a health risk to the subject.

9.2.3. Assessment of seriousness

The investigator is responsible for assessing the seriousness (serious or non-serious). If the incident is considered serious, this should be reported as a serious adverse event (SAE) by the investigator to the sponsor. See also section 9.3.2, Reporting of Serious Adverse Events (SAE).

9.3. Reporting and registration of Adverse Events

At each study visit, adverse events (AE) are registered, starting from start of the observation period, up to end of study. All AE that occurs during the study and which are observed by the investigator/study nurse or reported by the subject will be registered in the CRF regardless of whether they are related to the investigational product or not. Assessment of causal relationship, severity, and whether the AE is considered to be an SAE or not will be done by the investigator directly in eCRF. At minimum, for each AE/SAE, a description of the event is recorded (diagnosis/symptom if diagnosis is missing), start and stop dates, causal relationship, severity, if the AE is considered to be an SAE or not, measures and outcome.

Changes in the patients' medical condition that are consistent with natural disease progression are NOT considered as AEs and will be recorded as deterioration of the disease and not as AE in the medical record of the patient. Similarly, all medical conditions that are present at baseline will not be considered as AEs unless a worsening has occurred. In cases of surgical or diagnostic procedures, the condition/illness leading to such a procedure is considered as the AE rather than the procedure itself. In case of a fatality, the cause of death is considered as the AE.

9.3.1. Reporting of Adverse Events (AE)

All AE shall be registered in the CRF within a week.

9.3.2. Reporting of Serious Adverse Events (SAE)

Serious adverse events (SAE) are reported to the sponsor, via Kliniskt Forskningscentrum Umeå, on a SAE form within 24 hours of the investigator being informed of the SAE. The investigator determines if the event meets the protocol definition of an SAE, assesses causal relationship and intensity of the event. All SAE are reported to the sponsor independent of causality. The sponsor decides if the SAE fulfills the SUSAR criteria, and if so, is responsible for notifying the regulatory authorities.

Follow-up information describing the outcome and handling of the SAE is reported as soon as this information is available. The original should be kept in the Investigator Site File.

9.3.3. Reporting of Suspected Unexpected Serious Adverse Reactions (SUSAR)

Those SAE which are assessed by sponsor to be SUSAR are reported via a CIOMS form to the European Medicines Agency (EudraVigilance database) according to the specified time frames.

SUSAR that are fatal or life-threatening are reported as soon as possible and no later than 7 days after the incident has become known to the sponsor. Relevant follow-up information is sent thereafter within an additional 8 days. Other SUSAR are reported as soon as possible and no later than 15 days after they have come to the sponsor's knowledge.

Information about SUSAR occurring during the study is compiled by the sponsor and sent out to the principal investigator at all participating centers.

9.4. Follow-up of Adverse Events

All reported SAEs that have not been resolved by the end of the study will be followed up until the event has subsided (or disappeared), the condition has stabilized, the event is otherwise explained, or the study subject is lost to follow-up. SAE occurring after trial termination must be reported if considered related to the treatment during the trial.

9.5. Annual Safety Report (Development Safety Update Report, DSUR)

A yearly safety report will be sent to the Swedish Medical Products Agency and the Swedish Ethical Review Authority. The safety report will summarize all serious adverse events recorded during the year, as well as a summary assessment of the safety of patients and if the risk-benefit assessment has changed.

9.6. Procedures in case of emergencies, overdose or pregnancy

The sponsor and investigator will immediately take the urgent safety measures necessary to protect the subjects from immediate danger. The sponsor will inform the Swedish Medical Products Agency and EPM as soon as possible about the urgent safety measures taken by the investigator or sponsor.

If a study subject who participates in the trial becomes pregnant, this person must be followed up until the birth has taken place. If the fetus/child has any congenital malformation, this must be reported as a serious adverse event or side effect (SAE).

9.7. Reference Safety Information

A Summary of Product Characteristic (or SmPC)for Dapagliflozin will be used as Product Reference Safety Information.

10. Statistics

With multiple observations for each individual in three different time periods we will need to analyse both the difference of the values in the different time periods as well as the slope of change within the time periods as both the absolute values and the slope of change may differ.

10.1. Model the difference in outcome between periods

A mixed effect model will be used to model the difference between longitudinal measures in different periods. The model includes period as the fixed effect and individual as the random effect. $yij=\beta 0+\beta 1xij+ui+\varepsilon ij$
In the equation, y is the continuous outcome; x is the period; i is the individual and j is the longitudinal visits; $\beta 0$ is the estimated outcome level in baseline period; $\beta 1$ the estimated difference in outcome levels between different periods and the baseline; u is the random intercept for each individual and it follows a normal distribution; ε is the random error.

10.2. Model the difference and the change in outcome over time between periods

A piecewise linear mixed model will be used to estimate both the mean outcome level and the change of outcome over time in each period. In addition to the base model, we add the time and the interaction between time and period as fixed effects to the model.
$yij=\beta 0+\beta 1xij+\beta 2tij+\beta 3tijxij+ui+\varepsilon ij$
In the equation, t is the follow-up time in week; $\beta 2$ Is the slope of the change in outcome over time in baseline period; $\beta 3$ is the difference in slopes between different periods.

Since this is a pilot study with the aim of providing data for the design of a larger study and is neither blinded nor randomised we will not be able to provide a power calculation and the results from the statistical calculations will be descriptive without p-values.
intercept for each individual and it follows a normal distribution; ε is the random error.

10.3. Statistical analysis population

Endpoints for clinical efficacy will be calculated for all patients who complete at least the two first study periods – the baseline period without treatment and the 12-week period with active treatment. Safety endpoints and AE will be presented for all test subjects completing the screening visit.

11. Quality Control and Quality Assurance

11.1. Quality Assurance and Sponsor oversight

It is the investigators responsibility that the CRFs are reviewed for completeness and accuracy. It is also the investigators responsibility to ensure compliance with GCP and all applicable regulatory requirements. Regulatory agencies may conduct a regulatory inspection of this trial. Such audits/inspections can occur at any time during or after completion of the trial. If an audit or inspection occurs, the investigator and institution agree to allow the audit/inspection.

11.2. Monitoring

The study will be monitored by an independent monitor before the study begins, during the study conduct, and after the study has been completed, to ensure that the study is carried out according to the protocol and that data is collected, documented, and reported according to ICH-GCP and applicable ethical and regulatory requirements. Monitoring is performed as per the study's monitoring plan and is intended to ensure that the subject's rights, safety, and well-being are met as well as data in the CRF are complete, correct, and consistent with the source data.

11.3. Source data

The investigator must keep source documents for each subject in the study. A document describing what has been classified as source data in the study should be included in the Investigator Site File (ISF). The investigator must ensure that all source documents are accessible for monitoring and other quality control activities.

Source data is defined before study start at site.

11.4. Deviations or serious breaches

Serious breaches and deviations from the study protocol, GCP and other regulations that significantly and directly affects, or with high likelihood could affect, the subjects in Sweden or the scientific value of the study, shall be immediately reported within 7 days (from knowledge) to the Swedish Medical Products Agency. It is the sponsor's responsibility to judge the consequences of deviations that have occurred, and thus also to decide whether the Swedish Medical Products Agency should be informed.

Minor deviations that do not affect subjects' integrity or safety, nor significantly affect the study's scientific value, are documented in the study documentation of the principal investigator and the sponsor.

11.5. Inspections

Authorized representatives for Competent Authorities (CA) may carry out inspections at the study site, including source data verification. The investigator must ensure that all source documents are available for audits and inspections. The purpose of an inspection is to review all study-related activities and documents systematically and independently, so as to determine whether these activities were performed, registered, analyzed and reported correctly according to protocol, Good Clinical Practice (GCP) and applicable regulations.

12. Ethics

12.1. Compliance to the protocol, GCP and regulations

The study will be performed in compliance with the study protocol, the Declaration of Helsinki, ICH-GCP (Good Clinical Practice) guidelines and current national and international regulations governing this clinical trial. This is to ensure the safety and integrity of the study subjects as well as the quality of the data collected.

12.2. Ethical review of the study

The final study protocol for clinical trials must be approved, as a part of the application for a permit for clinical trials, by both the Swedish Ethical Review Authority (Etikprövningsmyndigheten, EPM) and the Swedish Medical Products Agency before the trial can be conducted. The final version of the informed consent form and other information provided to subjects, must be approved or given a written positive opinion by EPM. EPM and

the Swedish Medical Products Agency must be informed of any changes in the study protocol in accordance with current requirements.

12.3. Procedure for obtaining informed consent

The principal investigator at each site shall ensure that the subject is given full and adequate oral and written information about the study, its purpose, any risks and benefits as well as inclusion and exclusion criteria. Subjects must also be informed that they are free to discontinue their participation in the study at any time without having to provide a reason. Subjects should be given the opportunity to ask questions and be allowed time to consider the provided information. If the person chooses to participate, both the subject and the investigator shall sign the informed consent form. A copy of the subject information as well as the informed consent form shall be provided to the subject. The subject's signed and dated informed consent must be obtained before performing any study-specific activity in the study. Each subject who participated in the study will be identified by a subject number on a subject identification list. The subject agrees that monitors, auditors, and inspectors may have access to their medical records and other source data. If new information is added to the study, the subject has the right to reconsider whether he/she will continue their participation.

12.4. Data protection

If any part of the data is handled by any other organization, inside or outside the EU, appropriate agreements and/or other documentation will be established, to ensure that the data processing is performed in accordance with the provisions of the General Data Protection Regulation (GDPR) and other relevant legislation before any data transfer takes place.

The content of the informed consent form complies with relevant integrity and data protection legislation. In the subject information and the informed consent form, the subject will be given complete information about how collection, use and publication of their study data will take place. The subject information and the informed consent form will explain how study data are stored to maintain confidentiality in accordance with national data legislation. All information processed by the sponsor will be pseudonymized and identified with Study ID.

The informed consent form will also explain that for verification of the data, authorized representatives of the sponsor, as well as relevant authority, may require access to parts of medical records or study records that are relevant to the study, including the subject's medical history.

12.5. Insurances

Study subjects are insured through Swedish patient insurance as well as the Swedish pharmaceutical insurance.

13. Substantial changes to the study

Substantial changes to the signed study protocol are only possible through approved protocol amendments and by agreement from all responsible persons. Information on non-substantial changes should be clearly noted in the amended protocol.

In the event that substantial changes to the protocol (e.g., changing of the main objective, primary or secondary variables, method to measure the primary variable, changing of the investigational product or dosage) will be made during the course of the study, approval from the Swedish Ethical Review Authority (Etikprövningsmyndigheten, EPM) as well as the Swedish Medical Products Agency (Läkemedelsverket) shall be obtained before any changes are implemented. A change that concerns a new site, new investigator or a new study patient information sheet shall only be approved by EPM.

Non-substantial changes will be recorded and later entered in documentation that is submitted, for example in any subsequent notifications of a substantial change or in connection with End of Trial reporting.

The investigator must not make any deviation from or change of the protocol, except which necessary to eliminate an immediate risk to the study subjects.

14. Collection, handling, and archiving data

Subjects who participate in the study are coded with a specific study identification number. All subjects are registered in a subject identification list (subject enrolment and identification list) that connects the subject's name and personal number with a study identification number.

All data will be registered, managed, and stored in a manner that enables correct reporting, interpretation, and verification. The complete Trial Master File, as well as source documents, will be archived for at least 10 years according to EU-directive 2001/20/EG after the study is completed. Source data in the medical records system is stored and archived in accordance with the respective hospital regulations.

14.1. Case Report Form

A paper Case Report Form (CRF) for each participating subject is used for data collection and collected in subject specific binders. The investigator must ensure that data is registered and any corrections in the CRF are made as stated in the study protocol and in accordance with the instructions. The investigator must ensure that the registered data is correct, complete, and that reporting takes place according to the timelines that have been predefined and agreed. The investigator signs the completed CRF. A copy of the completed CRF will be archived at the study site.

If an examination/test is not performed and data does not exist, ND (Not done) or NK (Not known) is marked. If the question is irrelevant NA (Not applicable) is written. Corrections in

the paper CRF are done by striking out the incorrect information and adding the correct information next to the incorrect information, signing, and dating the correction.

15. Notification of study completion, reporting, and publication

The Swedish Medical Products Agency shall be informed of the study's completion at latest 90 days after study end, through submission of a ”Declaration of End of Trial Notification” form.

Within one year after the study is completed, the results shall be analyzed, a clinical study report with individual data shall be prepared, and the study results shall also be reported in the EudraCT database.

16. References

Adams, D., A. Gonzalez-Duarte, W. D. O'Riordan, C. C. Yang, M. Ueda, A. V. Kristen, I. Tournev, H. H. Schmidt, T. Coelho, J. L. Berk, K. P. Lin, G. Vita, S. Attarian, V. Planté-Bordeneuve, M. M. Mezei, J. M. Campistol, J. Buades, T. H. Brannagan, B. J. Kim, J. Oh, Y. Parman, Y. Sekijima, P. N. Hawkins, S. D. Solomon, M. Polydefkis, P. J. Dyck, P. J. Gandhi, S. Goyal, J. Chen, A. L. Strahs, S. V. Nochur, M. T. Sweetser, P. P. Garg, A. K. Vaishnaw, J. A. Gollob & O. B. Suhr (2018) Patisiran, an RNAi Therapeutic, for Hereditary Transthyretin Amyloidosis. N Engl J Med, 379, 11-21.

Aus dem Siepen, F., S. Hein, R. Bauer, H. A. Katus & A. V. Kristen (2017) Standard heart failure medication in cardiac transthyretin amyloidosis: useful or harmful? Amyloid, 24, 132-133.

Bonner, C., J. Kerr-Conte, V. Gmyr, G. Queniat, E. Moerman, J. Thévenet, C. Beaucamps, N. Delalleau, I. Popescu, W. J. Malaisse, A. Sener, B. Deprez, A. Abderrahmani, B. Staels & F. Pattou (2015) Inhibition of the glucose transporter SGLT2 with dapagliflozin in pancreatic alpha cells triggers glucagon secretion. Nat Med, 21, 512-7.

Bruins, S., M. R. Fokkema, J. W. Römer, M. J. Dejongste, F. P. van der Dijs, J. M. van den Ouweland & F. A. Muskiet (2004) High intraindividual variation of B-type natriuretic peptide (BNP) and amino-terminal proBNP in patients with stable chronic heart failure. Clin Chem, 50, 2052-8.

Ferrannini, E., M. Mark & E. Mayoux (2016) CV Protection in the EMPA-REG OUTCOME Trial: A "Thrifty Substrate" Hypothesis. Diabetes Care, 39, 1108-14.

Garcia-Pavia P, Rapezzi C, Adler Y, Arad M, Basso C, Brucato A, et al. Diagnosis and treatment of cardiac amyloidosis: a position statement of the ESC Working Group on Myocardial and Pericardial Diseases. Eur Heart J. 2021;42:1554

Grogan, M., C. G. Scott, R. A. Kyle, S. R. Zeldenrust, M. A. Gertz, G. Lin, K. W. Klarich, W. L. Miller, J. J. Maleszewski & A. Dispenzieri (2016) Natural History of Wild-Type

Transthyretin Cardiac Amyloidosis and Risk Stratification Using a Novel Staging System. J Am Coll Cardiol, 68, 1014-20.

Kang, S., S. Verma, A. F. Hassanabad, G. Teng, D. D. Belke, J. A. Dundas, D. G. Guzzardi, D. A. Svystonyuk, S. S. Pattar, D. S. J. Park, J. D. Turnbull, H. J. Duff, L. A. Tibbles, R. H. Cunnington, J. R. B. Dyck & P. W. M. Fedak (2020) Direct Effects of Empagliflozin on Extracellular Matrix Remodelling in Human Cardiac Myofibroblasts: Novel Translational Clues to Explain EMPA-REG OUTCOME Results. Can J Cardiol, 36, 543-553.

Lindmark, K., B. Pilebro, T. Sundström & P. Lindqvist (2021) Prevalence of wild type transtyrethin cardiac amyloidosis in a heart failure clinic. ESC Heart Fail, 8, 745-749.

Maurer, M. S., J. H. Schwartz, B. Gundapaneni, P. M. Elliott, G. Merlini, M. Waddington-Cruz, A. V. Kristen, M. Grogan, R. Witteles, T. Damy, B. M. Drachman, S. J. Shah, M. Hanna, D. P. Judge, A. I. Barsdorf, P. Huber, T. A. Patterson, S. Riley, J. Schumacher, M. Stewart, M. B. Sultan, C. Rapezzi & A.-A. S. Investigators (2018) Tafamidis Treatment for Patients with Transthyretin Amyloid Cardiomyopathy. N Engl J Med, 379, 1007-1016.

McMurray, J. J. V., S. D. Solomon, S. E. Inzucchi, L. Køber, M. N. Kosiborod, F. A. Martinez, P. Ponikowski, M. S. Sabatine, I. S. Anand, J. Bělohlávek, M. Böhm, C. E. Chiang, V. K. Chopra, R. A. de Boer, A. S. Desai, M. Diez, J. Drozdz, A. Dukát, J. Ge, J. G. Howlett, T. Katova, M. Kitakaze, C. E. A. Ljungman, B. Merkely, J. C. Nicolau, E. O'Meara, M. C. Petrie, P. N. Vinh, M. Schou, S. Tereshchenko, S. Verma, C. Held, D. L. DeMets, K. F. Docherty, P. S. Jhund, O. Bengtsson, M. Sjöstrand, A. M. Langkilde & D.-H. T. C. a. Investigators (2019) Dapagliflozin in Patients with Heart Failure and Reduced Ejection Fraction. N Engl J Med, 381, 1995-2008.

Neal B, Perkovic V, Mahaffey KW, de Zeeuw D, Fulcher G, Erondu N, et al, for the CANVAS Program Collaborative Group. Canagliflozin and cardiovascular and renal events in type 2 diabetes. N Engl J Med, 377:644-57

Packer, M., S. D. Anker, J. Butler, G. Filippatos, S. J. Pocock, P. Carson, J. Januzzi, S. Verma, H. Tsutsui, M. Brueckmann, W. Jamal, K. Kimura, J. Schnee, C. Zeller, D. Cotton, E. Bocchi, M. Böhm, D. J. Choi, V. Chopra, E. Chuquiure, N. Giannetti, S. Janssens, J. Zhang, J. R. Gonzalez Juanatey, S. Kaul, H. P. Brunner-La Rocca, B. Merkely, S. J. Nicholls, S. Perrone, I. Pina, P. Ponikowski, N. Sattar, M. Senni, M. F. Seronde, J. Spinar, I. Squire, S. Taddei, C. Wanner, F. Zannad & E.-R. T. Investigators (2020) Cardiovascular and Renal Outcomes with Empagliflozin in Heart Failure. N Engl J Med, 383, 1413-1424.

Perugini, E., P. L. Guidalotti, F. Salvi, R. M. Cooke, C. Pettinato, L. Riva, O. Leone, M. Farsad, P. Ciliberti, L. Bacchi-Reggiani, F. Fallani, A. Branzi & C. Rapezzi (2005) Noninvasive etiologic diagnosis of cardiac amyloidosis using 99mTc-3,3-diphosphono-1,2-propanodicarboxylic acid scintigraphy. J Am Coll Cardiol, 46, 1076-84.

Ponikowski, P., A. A. Voors, S. D. Anker, H. Bueno, J. G. F. Cleland, A. J. S. Coats, V. Falk, J. R. González-Juanatey, V. P. Harjola, E. A. Jankowska, M. Jessup, C. Linde, P. Nihoyannopoulos, J. T. Parissis, B. Pieske, J. P. Riley, G. M. C. Rosano, L. M. Ruilope, F.

Ruschitzka, F. H. Rutten & P. van der Meer (2016) 2016 ESC Guidelines for the Diagnosis and Treatment of Acute and Chronic Heart Failure. Rev Esp Cardiol (Engl Ed), 69, 1167.

Ruberg, F. L., M. Grogan, M. Hanna, J. W. Kelly & M. S. Maurer (2019) Transthyretin Amyloid Cardiomyopathy: JACC State-of-the-Art Review. J Am Coll Cardiol, 73, 2872-2891.

Solini, A., L. Giannini, M. Seghieri, E. Vitolo, S. Taddei, L. Ghiadoni & R. M. Bruno (2017) Dapagliflozin acutely improves endothelial dysfunction, reduces aortic stiffness and renal resistive index in type 2 diabetic patients: a pilot study. Cardiovasc Diabetol, 16, 138.

Suhr, O. B., J. Wixner, B. Pilebro, H. E. Lundgren & I. Anan (2017) The Swedish landscape of hereditary ATTR amyloidosis. Amyloid, 24, 93-94.

Verma, S., S. Rawat, K. L. Ho, C. S. Wagg, L. Zhang, H. Teoh, J. E. Dyck, G. M. Uddin, G. Y. Oudit, E. Mayoux, M. Lehrke, N. Marx & G. D. Lopaschuk (2018) Empagliflozin Increases Cardiac Energy Production in Diabetes: Novel Translational Insights Into the Heart Failure Benefits of SGLT2 Inhibitors. JACC Basic Transl Sci, 3, 575-587.

Westermark, P., J. Bergström, A. Solomon, C. Murphy & K. Sletten (2003) Transthyretin-derived senile systemic amyloidosis: clinicopathologic and structural considerations. Amyloid, 10 Suppl 1, 48-54.

Westermark, P., K. Sletten, B. Johansson & G. G. Cornwell (1990) Fibril in senile systemic amyloidosis is derived from normal transthyretin. Proc Natl Acad Sci U S A, 87, 2843-5.

17. Attachments

1. Kansas City Cardiomyopathy Questionnaire (KCCQ)
2. Karnofsky Performance Scale
3. Summary of Product Characteristics for Forxiga®
