# Supplementary material for: Using dapagliflozin to reduce symptoms in transthyretin cardiac amyloidosis: An n-of-1 trial series pilot study
Source: PLoS One. 2026 Oct 7;21(10):e0346065. doi: 10.1371/journal.pone.0346065 (PMC13645067; doi:10.1371/journal.pone.0346065)
Supplement: Supplementary file 2 [file pone.0346065.s002.docx]

# Statistical analysis plan

With multiple observations for each individual in three different time periods we will need to analyse both the difference of the values in the different time periods as well as the slope of change within the time periods as both the absolute values and the slope of change may differ.

Model the difference in outcome between periods

A mixed effect model will be used to model the difference between longitudinal measures in different periods. The model includes period as the fixed effect and individual as the random effect. 𝑦_𝑖𝑗_=𝛽_0_+𝛽_1𝑥𝑖𝑗_+𝑢_𝑖_+𝜀_𝑖𝑗_ In the equation, 𝑦 is the continuous outcome; 𝑥 is the period; 𝑖 is the individual and 𝑗 is the longitudinal visits; 𝛽_0_ is the estimated outcome level in baseline period; 𝛽_1_ the estimated difference in outcome levels between different periods and the baseline; 𝑢 is the random intercept for each individual and it follows a normal distribution; 𝜀 is the random error.

Model the difference and the change in outcome over time between periods

A piecewise linear mixed model will be used to estimate both the mean outcome level and the change of outcome over time in each period. In addition to the base model, we add the time and the interaction between time and period as fixed effects to the model. � y_𝑖𝑗_=𝛽_0_+𝛽_1𝑥𝑖𝑗_+𝛽_2𝑡𝑖𝑗_+𝛽_3𝑡𝑖𝑗𝑥𝑖𝑗_+𝑢_𝑖_+𝜀_𝑖𝑗_ In the equation, 𝑡 is the follow-up time in week; 𝛽2 Is the slope of the change in outcome over time in baseline period; 𝛽3 is the difference in slopes between different periods.

Since this is a pilot study with the aim of providing data for the design of a larger study and is neither blinded nor randomised we will not be able to provide a power calculation and the results from the statistical calculations will be descriptive without p-values.

## Statistical analysis population

Endpoints for clinical efficacy will be calculated for all patients who complete at least the two first study periods – the baseline period without treatment and the 12-week period with active treatment. Safety endpoints and AE will be presented for all test subjects completing the screening visit.
