# Supplementary material for: Using dapagliflozin to reduce symptoms in transthyretin cardiac amyloidosis: An n-of-1 trial series pilot study
Source: PLoS One. 2026 Oct 7;21(10):e0346065. doi: 10.1371/journal.pone.0346065 (PMC13645067; doi:10.1371/journal.pone.0346065)
Supplement: Supplementary file 3 [file pone.0346065.s003.docx]

**Supplementary Table 2. Pairwise estimated marginal mean differences from exploratory model 2**

Exploratory mixed-effects model including study period, week, and period-by-week interaction terms. Values are based on estimated marginal means. P-values are descriptive only and were not used for confirmatory inference.

| **Outcome** | **Comparison** | **Mean difference** | **SE** | **df** | **p-value** | **95% CI** |
| --- | --- | --- | --- | --- | --- | --- |
| NT-proBNP | Baseline vs intervention | 521.2 | 240.7 | 69.7 | 0.034 | 41.0 to 1001.4 |
|  | Baseline vs withdrawal | 298.1 | 346.6 | 50.8 | 0.394 | -397.8 to 993.9 |
|  | Intervention vs withdrawal | -223.1 | 240.7 | 69.7 | 0.357 | -703.3 to 257.0 |
| 6MWT | Baseline vs intervention | -24.2 | 29.4 | 48.0 | 0.415 | -83.4 to 35.0 |
|  | Baseline vs withdrawal | -17.7 | 41.5 | 42.3 | 0.671 | -101.5 to 66.0 |
|  | Intervention vs withdrawal | 6.5 | 29.4 | 47.2 | 0.826 | -52.6 to 65.6 |
| KCCQ-TSS | Baseline vs intervention | 1.4 | 4.5 | 49.5 | 0.761 | -7.6 to 10.3 |
|  | Baseline vs withdrawal | -1.1 | 6.5 | 45.3 | 0.865 | -14.1 to 11.9 |
|  | Intervention vs withdrawal | -2.5 | 4.4 | 50.0 | 0.577 | -11.3 to 6.4 |

**Abbreviations:** SE, standard error; CI, confidence interval; NT-proBNP, N-terminal pro-brain natriuretic peptide; 6MWT, 6-minute walking test; KCCQ-TSS, Kansas City Cardiomyopathy Questionnaire total symptom score.

**Note:** Mean differences are expressed as first period minus second period. Estimates are shown to one decimal place for readability. The p-values are unadjusted least significant difference comparisons from the exploratory model and should be interpreted descriptively.
